# An Update on the Role of Arterial Stiffness in Heart Failure and the Treatment of Dyslipidemia

**DOI:** 10.3390/jcm12093270

**Published:** 2023-05-04

**Authors:** Mauro Feola

**Affiliations:** Department of Cardiology, Ospedale Regina Montis Regalis, Strada del Rocchetto 99, 12084 Cuneo, Italy; m_feola@virgilio.it; Tel.: +39-0174-677730; Fax: +39-0174-677306

Vascular aging in large arteries plays an important role in contributing to cardiovascular morbidity and mortality [1]. The sub-clinical atherosclerosis of arteries determines their structural changes, including increases in their wall thickness; the intima-media thickening ratio seems to be independently correlated with cardiovascular prognoses [1]. In large arteries, according to aging, their main functional modification consists of a real “stiffening”. Arterial stiffness, assessed using pulse wave velocity (PWV), has proved to be a strong independent predictor of cardiovascular prognoses [1]; it plays an important role in terms of age-related increases in systolic blood pressure and pulse pressure [2], which are both components of blood pressure that are closely associated with cardiovascular risks in middle-aged or elderly subjects.

While the thickening of the arterial walls should be much more correlated with atherosclerotic processes, arterial stiffening aetiology should be linked to degenerative/calcified processes. However, the mechanisms through which both of these processes are related have yet to be clarified. Recently, the Consensus Document [3] on ventricular–arterial coupling in cardiac disease recognized PWV as the gold standard for non-invasive examination that is able to study large arterial stiffness. Moreover, the document analyzed the role of arterial stiffness in heart failure with reduced ejection fraction (HFrEF) and heart failure with preserved ejection fraction (HFpEF), suggesting the measurement of ventricular–arterial coupling to be one important parameter for the management of its therapy. Its possible pathogenetic role and influence on the clinical prognosis of arterial stiffness in heart failure patients is still under debate.

In the recent literature, there has been emerging interest in the influence of the parameters that reflect the aortic stiffness of patients suffering with HF. In research on the possible effect of PWV on HF [4], we documented in 59 HF patients that PWV proved to be lower in comparison with the cardiovascular risk factor (CVRF) population. Furthermore, in comparison to HF patients, the CVRF subjects proved to have a higher brachial SP and central SP that may have influenced the PWV values, confirming that central/brachial BP and PWV are closely related. Large artery stiffness, influenced by aging, diabetes mellitus, atherosclerosis, and renal failure, is the main determinant of pulsatory pressure (PP). The central/brachial PP did not prove to be different between the three populations examined and should not be considered as a marker of aortic elasticity for its dependence on left ventricular function.

Moreover, the values of augmentation index (AIx75) in the HF group vs. the CVRF and healthy population seemed to be different (22% vs. 34% and 32%, respectively), demonstrating significantly lower values for the HF group. It can be hypothesized that a reduction/delay in the arrival of the reflected wave can enter into the determinism of the left ventricular remodeling, since the arrival of the reflected wave creates an additional obstacle to the ejection of the left ventricle. Furthermore, the statistical analysis demonstrated that the correlation between the values of PWV/AIx75 and the value of the renal filtrate (GFR) was significantly the strongest, as underlying renal dysfunction might determine an important effect that increases the aortic stiffness, which could be crucial in the evolution of both heart failure and vascular damage. More recently [5], we analyzed 199 HF patients that were discharged alive after an acute decompensation episode, reviewing the prognostic power of the arterial stiffness parameters in 14-month follow-up. After the adjustment for the principal confounders, the PWV, Aix75, and stiffness index proved to be inversely correlated with free-event survival (death and rehospitalisation for HF) (*p* = 0.006, *p* < 0.001, and *p* < 0.0001, respectively). In particular, a PWV pre-discharge value of ≥ 10 m/s was inversely correlated with free-event survival (HR = 1.7, *p* = 0.03). These data suggest that non-invasive measurements of arterial stiffness in HF patients discharged alive after an acute decompensation might be considered as useful prognostic parameters [6].

Alirocumab, a human monoclonal antibody to proprotein convertase subtilisin–kexin type 9 (PCSK9), improved the cardiovascular outcomes after an acute coronary syndrome in patients receiving high-intensity statin therapy and clearly reduced the plasma level of their LDL cholesterol after a 24-month treatment [7]. Upon treating ischemic and dyslipidemic patients with alirocumab, at a 6-month follow-up, we observed a decline in their LDL cholesterol from 140.3 ± 64.9 mg/dL to 73.6 ± 15.5 mg/dL (*p* = 0.03), together with a reduction in their PWVs from 13.7 ± 2.4 m/s to 10.5 ± 1.43 m/s (*p* < 0.05) [8]. A linear correlation between the total cholesterol or LDL cholesterol and arterial stiffness has been correctly proved [9] and the reduction with the statins of LDL cholesterol usually reduced the PWV in the treated patients. The interesting question is if the arterial stiffness proved to be increased in familial hypercholesterolemia (FH), an in vivo model of life-long exposure to an elevated level of plasma cholesterolemia, which is the most important risk factor for accelerated and premature atherosclerotic cardiovascular disease. The question of if FH patients have augmented arterial stiffness parameters or not is under debate. Furthermore, in 30 male FH patients followed for 6 months, the administration of PCSK9 inhibitors (alirocumab/evolocumab) determined a reduction of 48% in their LDL cholesterol and significantly improved their PWVs from 9.86 ± 1.5 m/s to 7.7 ± 1.42 m/s (*p* < 0.05) [10]. This real-world experience demonstrated that, in a young FH population, the main result seemed to be a decline in arterial stiffness correlated with a huge reduction in LDL cholesterol, even if the prognostic meaning of this modification needs to be evaluated in a prognostic study.

In conclusion, the analysis of arterial stiffness seemed to be promising for the determinism and prognosis of HF patients. In the search for a prognostic meaning in HF patients, aortic stiffness seems to be particularly related to renal dysfunction and is extremely important in the prognostic setting. Finally, the huge reduction in LDL cholesterol obtained with PCSK9 inhibitors might influence the arterial stiffness of treated patients, even in a short/mid-term follow-up.
